# Similar strength gains at lower perceived efforts via cluster set vs. traditional home-based online training: A 6 weeks randomized controlled trial

**DOI:** 10.3389/fspor.2022.968258

**Published:** 2022-08-25

**Authors:** Ludwig Rappelt, Steffen Held, Mario Leicht, Pamela Wicker, Lars Donath

**Affiliations:** ^1^Department of Intervention Research in Exercise Training, German Sport University Cologne, Cologne, Germany; ^2^Department of Sports Science, Bielefeld University, Bielefeld, Germany

**Keywords:** inter-repetition rest training, bodyweight training, elderly, tele training, health, balance

## Abstract

Cluster Training (CT) has been shown to induce strength at lower perceived efforts compared to traditional training (TRT) with sets performed to repetition failure. These findings have not yet been extended to remote online training in middle-aged to older people. Thus the present study aimed at investigating whether a cluster set online training with bodyweight exercises is similar in its effectiveness a more demanding traditional strength training employed with a traditional set structure. A total of *n* = 21 participants (14 female, 55 ± 12 years, 76.4 ± 16.1 kg, 1.71 ± 0.10 m, 74 ± 72 min of activity/w) were randomly assigned to either a CT or volume-, load-, and work-to-rest-ratio-matched TRT. After an initial 6-week run-in-phase, all participants were engaged into an online live-instructed full-body workout twice a week (40 min each) for a period of 6 weeks. Rates of perceived efforts (RPE) were assessed for each session (session RPE; sRPE). Changes in maximal voluntary contraction (MVC) at leg press (LP) and abdominal press (AP) as well as one-minute-sit-to-stand and Y-Balance-Test (YBT) were compared between BASELINE and PRE (ΔRUN-IN) and between PRE and POST (ΔINTERVENTION). In LP, TRT showed greater improvements with large effect sizes in ΔINTERVENTION compared to ΔRUN-IN. In CT, greater improvements with moderate effects were found in ΔINTERVENTION compared to ΔRUN-IN. In AP, both CT and TRT showed larger improvements with large effect sizes in ΔINTERVENTION compared to ΔRUN-IN. In YBT, a significant and large main effect for time was found indicating larger improvements for ΔINTERVENTION compared to ΔRUN-IN. CT showed lower sRPE than TRT. Both CT and TRT led to similar adaptations in MVC and balance performance. However, the perceived effort of CT was rated lower than for TRT. Therefore, conducting resistance training with a cluster set structure seems to be a suitable approach for training programs in middle-aged and older people.

## Introduction

One third of the Western population will be older than 65 years by the end of the current century (Lutz et al., 2008). The process of biological aging is accompanied with physical and cognitive deteriorations (Grady, 2012) that can lead to reductions in physical independence (Cress and Meyer, 2003). Regular physical activity and exercise training ranging from low to high intensities can diminish the risk of age-related declines of physical and cognitive dysfunction (McPhee et al., 2016) and increase the quality of life (McAuley et al., 2006). However, a majority of middle-aged women and men do not meet the physical activity level recommended (Mynarski et al., 2014). This insufficient level of physical activity is associated with health concerns at older age (Haapanen-Niemi et al., 2000) and an increased mortality rate (Byberg et al., 2009).

The COVID-19 pandemic and its partially associated social isolation (e.g., quarantines, isolation) strongly affected physical activity levels (Meyer et al., 2020). During home quarantine, 41.3% of citizens over 60 years showed prevalence of insufficient physical activity (Qin et al., 2020). However, although especially present during the pandemic, social isolation is considered a growing public health concern and health problem for older adults (Holt-Lunstad et al., 2015; Boulos et al., 2017). Socially isolated individuals (i.e., lacking social contact, having few relationships with other people and little involvement in social organizations; Holt-Lunstad et al., 2015; Schrempft et al., 2019) are more likely to be physically inactive (Delerue Matos et al., 2021). In this context, home-based training programs appear to be suitable means to attenuate the impact of physical inactivity because of the COVID-19 pandemic burden and other instances of social isolation. It has been shown that lower extremity strength and functional ability can be improved via home-based training programs in older adults (Thiebaud et al., 2014). However, the magnitude of potential improvements of static and dynamic balance as well as lower extremity strength and power have been linked to the extent and quality of supervision during the intervention period (Lacroix et al., 2017). Thus, regular face-to-face communication between coaches and participants during home-based fitness training using video calls seems promising to ensure compliant attendees and efficient training adaptations (Chan et al., 2021). The effects of a home-based, online, and live instructed strength training, however, have not yet gained much attention from a scientific perspective, particularly among seniors (Schwartz et al., 2021).

Cluster Training (CT) or inter-repetition rest training describes a training modality in which short, intra-set rest periods are implemented between the repetitions within one set (Latella et al., 2019). In a recently published meta-analysis, Davies and colleagues found similar improvements in muscular strength, power, and hypertrophy between traditional resistance training (TRT) and CT, while CT induced less fatigue (Davies et al., 2021). However, most of the included studies were conducted with young and physically active participants, leaving a need for research on the effects of such CT approaches in middle-aged and older individuals (Latella et al., 2019; Davies et al., 2021). Thus, differences in strength adaptations between training programs with a traditional set structure and a cluster set structure conducted as a homebased online live training have not yet been addressed in middle-aged to older adults.

The associations between intense exercise and discomfort were repeatedly reported by elderly people as barriers to engage in exercise (Franco et al., 2015). Especially TRT performed to repetition failure has been shown to induce high physical exhaustion levels with high rates of perceived effort (RPE), at least in young adults (Sánchez-Medina and González-Badillo, 2011; Fisher et al., 2016). In the elderly, fatigue of the trunk and lower extremities has been shown to lead to impaired balance ability (Helbostad et al., 2010) and is associated with a significant increase in the risk of falls (Helbostad et al., 2010; Nagano et al., 2014; Renner et al., 2021). Exercise which induces high fatigue, therefore, acutely presents a transient risk of falls for elders in the daily life (Donath et al., 2015).

Comparing CT configurations to work-load-matched TRT in both younger, strength-training-experienced adults (Guardado et al., 2021) and older healthy men (Dello Iacono et al., 2020) has led to lower RPE. It was speculated, that lower lactate levels and lower levels of muscle pain in turn led to a lower rating of perceived effort (Guardado et al., 2021). Moreover, for TRT a greater impulse and lower power output was found for the same number of repetitions per set (Dello Iacono et al., 2020). Therefore, conduction strength training in a CT design might be more suited for older individuals.

Therefore, this randomized control trial aimed at elucidating if similar strength and functional balance adaptations can be gained at a lower perceived effort after 6 weeks of homebased online live training with a cluster training set structure compared to a volume-matched traditional training set structure in healthy, untrained middle-aged to older adults. These results might impact the design of homebased training programs designed for seniors, as online training can be made available to a larger number of people at low cost and independent of location.

## Materials and methods

### Participants

Based on a previously published randomized controlled trial conducted with a comparable population (Dias et al., 2020), a power analysis (α = 0.05, study power [1-β-error] = 0.80, r = 0.8, effect size η_p_^2^ = 0.059 [*f* = 0.25]) performed using g^*^Power (Version 3.1.9.6, University of Kiel, Germany) required a sample size of *n* = 16. Assuming moderate to high rates of dropout, a total of *n* = 27 participants were recruited via social media and newspaper announces. Inclusion criteria were (I) aged between 40 and 70 years, (II) no medical conditions potentially impeding the completion of all testing and training sessions, (III) no acute infections, and (IV) no acute or chronic intake of medications such as analgesics, sleeping pills, antidepressants, and tranquilizers.

After the run-in-phase (lab visit II; see study design), all included participants were either assigned to a traditional training (TRT) or a cluster training design group (CT) using the minimization method (Scott et al., 2002). Age, body mass index (BMI), sex, mean isometric strength test results (in Newton, see testing procedure), mean activity per week (in minutes), and initial level of exercise difficulty (see training procedures) were used as strata for the minimization procedure. During the run-in phase, *n* = 2 participants dropped out due to personal reasons. Furthermore, *n* = 4 participants dropped out during the intervention due to personal (*n* = 2) and health related reasons (*n* = 1 with a cold and *n* = 1 with an ankle injury not related to the study). No further dropouts occurred. Hence, a total of *n* = 21 participants completed the study with at least 80% adherence to the training sessions and were therefore included in the final per-protocol analyses (Table 1). The study was approved by the local ethical committee of the German Sport University Cologne (37/2021) and all participants signed an informed written consent prior to start of the study.

**Table 1:** Mean values ± standard deviations for anthropometric data, performance indices and items of the SF-36 questionnaire of both cluster training (CT) and traditional training group (TRT) at PRE.

**Parameter**	**CT (*n* = 10)**	**TRT (*n* = 11)**	***p*-value**	** $\eta_{\text{p}}^{2}$ **	**SMD**
Sex [f/m]	6/4	8/3	–	–	–
Age [yrs]	54.2 ± 12.6	53.2 ± 11.1	*p* = 0.846	0.002	0.086
Mass [kg]	75.1 ± 14.9	77.8 ± 17.8	*p* = 0.715	0.007	0.162
Height [m]	1.71 ± 0.10	1.73 ± 0.10	*p* = 0.652	0.011	0.200
BMI [kg × m^−2^]	25.3 ± 2.6	25.7 ± 4.2	*p* = 0.763	0.005	0.134
Activity [min × w^−1^]	93.5 ± 71.8	55.4 ± 69.4	*p* = 0.232	0.074	0.540
YBT [cm]	243.1 ± 25.9	238.9 ± 27.6	*p* = 0.728	0.007	0.155
LP [N]	2,083 ± 515	2,041 ± 665	*p* = 0.875	0.001	0.070
AP [N]	704 ± 214	683 ± 189	*p* = 0.819	0.003	0.101
OM-STS [a.u.]	42.8 ± 10.9	38.6 ± 12.2	*p* = 0.421	0.034	0.360
PF [a.u.]	93.0 ± 8.6	90.5 ± 8.8	*p* = 0.510	0.023	0.293
RP [a.u.]	95.0 ± 10.5	84.1 ± 30.2	*p* = 0.292	0.058	0.473
BP [a.u.]	82.0 ± 19.3	75.5 ± 27.7	*p* = 0.541	0.020	0.272
GH [a.u.]	73.5 ± 15.6	66.8 ± 17.8	*p* = 0.374	0.042	0.398
VT [a.u.]	62.5 ± 13.0	54.1 ± 22.1	*p* = 0.308	0.550	0.458
SF [a.u.]	90.0 ± 11.5	84.1 ± 18.6	*p* = 0.398	0.038	0.378
RE [a.u.]	90.0 ± 22.5	72.7 ± 32.7	*p* = 0.179	0.093	0.609
MH [a.u.]	76.4 ± 9.7	68.7 ± 15.8	*p* = 0.201	0.085	0.579
PCS [a.u.]	85.9 ± 7.0	79.2 ± 18.8	*p* = 0.305	0.055	0.461
MCS [a.u.]	79.7 ± 9.2	69.9 ± 19.8	*p* = 0.168	0.097	0.626

p-values and partial eta squared ($\eta_{\text{p}}^{2}$) of 1 × 2 ANOVA are shown. Further, standardized mean differences (SMD) of pairwise comparison are indicated.

YBT, Y-Balance-Test; LP, Leg Press; AP, Abdominal Press; OM-STS, one-minute sit-to-stand-test; PF, physical functioning; RP, role limitations due to physical health problems; BP, bodily pain; GH, general health; VT, vitality; SF, social functioning; RE, role limitations due to emotional problems; MH, mental health; PCS, physical component of health; MCS, mental component of health.

### Design

**Figure 1:**
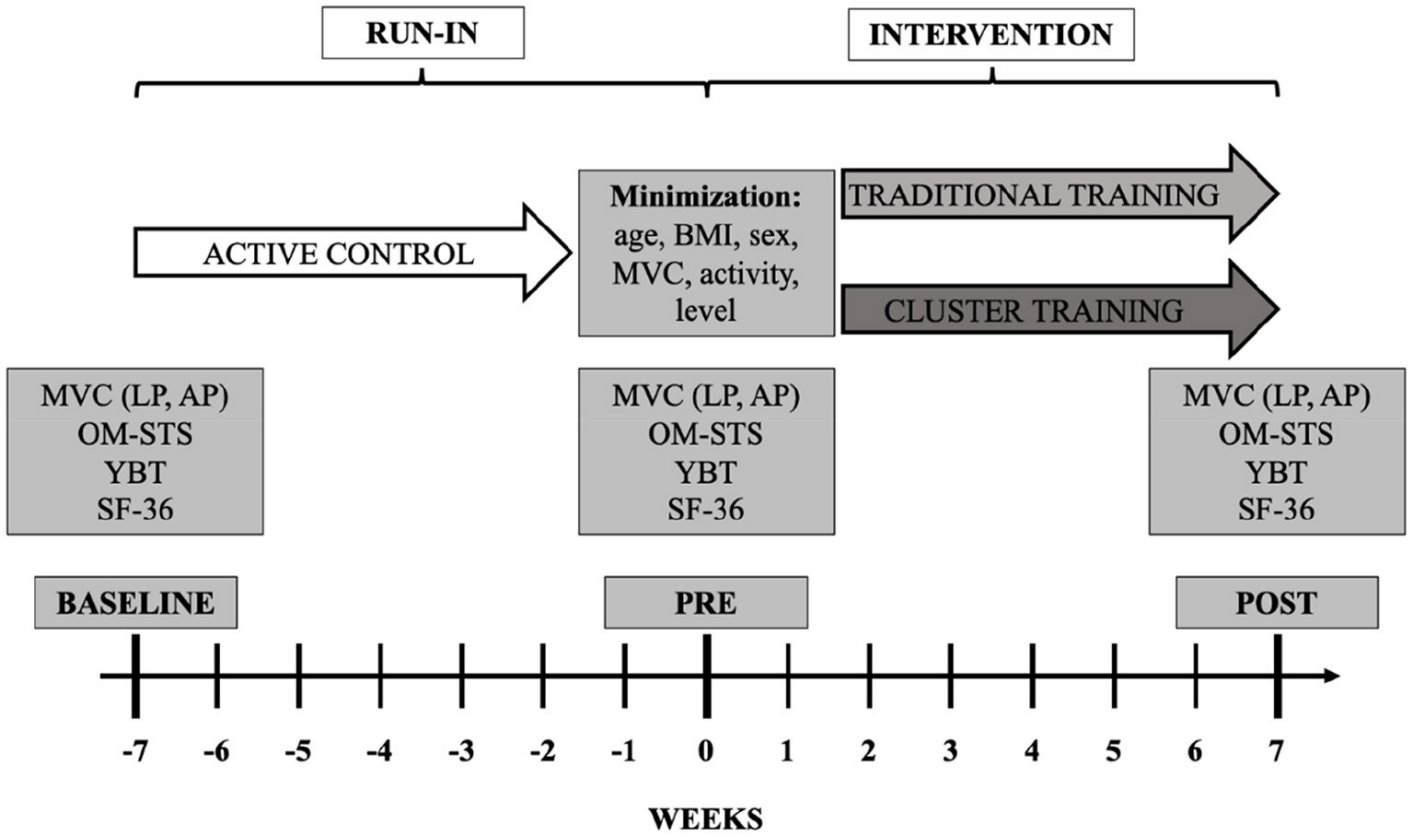
Schematic representation of the experimental protocol. Testing at BASELINE, PRE, and POST consisted of maximal isometric voluntary contraction (MVC) at leg press (LP) and abdominal press (AP), one-minute sit-to-stand (OM-STS), Y-Balance-test (YBT), and quality-of-life-questionnaire (SF-36). After PRE, group assignment was performed by employing the minimization method using age, body mass index (BMI), sex, maximal voluntary contraction of abdominal press and leg press in N (MVC), mean physical activity in minutes (activity), and exercise level (level) as strata.

The study was designed as a randomized two-group, parallel trial with a run-in phase. During the study, all participants completed a total of three lab visits at baseline (week−7), immediately before the start of the intervention (week 0), and immediately after the completion of the intervention program (week 7) (Figure 1). For all further analyses, the respective differences for all outcome measures between lab visit I and lab visit II (ΔRUN-IN) and between lab visit II and lab visit III (ΔINTERVENTION) were calculated, with values >0 indicating increases. During all lab visits, anthropometric data were collected before a short warm-up on the rowing ergometer, functional balance performance testing, isometric maximum strength assessment on the abdominal press and the leg press, and dynamic strength endurance evaluation of the lower extremities using a one-minute sit-to-stand-test, in the described order. Further, psychometric data (Quality of life assessment, SF-36 questionnaire; Kurth and Ellert, 2002) were examined. All lab visits were scheduled at the same time of day to avoid circadian influences within participants. The 6-week run-in phase between lab visit I and II was used as an active control period in which participants were instructed to maintain their normal habitual daily activity level. During the intervention phase between lab visits II and III, both groups were face-to-face coached for 6 weeks in a home-based online-training program setting, with training conducted twice a week live and video-based using the Webex platform (Cisco Systems, Inc., San José, California, USA).

### Testing procedures

Maximal voluntary contraction (MVC, in N) under isometric conditions was assessed at leg press (LP) and abdominal press (AP) machines (Edition-Line, gym80, Gelsenkirchen, Germany). These machines were equipped with strain gauge sensors attached in line with the steel belt lifting the machines weight plates (KM1506, megaTron, Munich, Germany), a PC-2-Channel-Interface, and a computer with the corresponding software recording at a rate of 100 Hz (IsoTest 2, mechaTronic, Hamm, Germany). For both leg and abdominal press, each participant performed three maximal isometric test attempts. Participants were instructed to reach the peak force as quickly as possible by pushing “hard and fast” (Maffiuletti et al., 2016). Isometric leg press testing was conducted in an upright sitting position at an inner knee angle of 120°. Isometric strength tests on abdominal press were also conducted in an upright sitting position with a hip angle of 90°. The highest consecutive force values averaged over 150 ms were calculated for each attempt. The average of the two best attempts of LP and AP were used for further analyses, respectively.

In order to assess the dynamic strength endurance ability of the lower extremities, a one-minute sit-to-stand test (OM-STS) was conducted. Participants were seated on a chair with no arm support. The height of the seat was standardized for all participants at 46 cm from the floor to the top of the seat. The participants were instructed to keep their feet positioned at the floor in front of the chair and their hands folded in front of their upper body while standing up and sitting down without using the arms for support as quickly as possible. For each repetition, it was ensured that balance was adequately achieved before sitting. The total number of sit-to-stand repetitions completed in 60 s was counted and used for further analyses. A high reliability was previously reported for the OM-STS test (ICC = 0.80) (Ritchie et al., 2005).

Functional balance performance was tested without shoes using the Y-balance-Test (Powden et al., 2019). This test is conducted using a Y-shaped plastic device (Functional Movement Systems, Chatham, USA). The aim of the test is to push a plastic box with one foot as far as possible in the anterior, posterior-medial, and posterior-lateral direction while remaining balanced on the other leg. Participants were instructed to place the hands on their hips, to only touch the box on the vertical surface, and to push not kick the box to the respective direction. During the whole test, participants had to maintain balance on their dominant leg. Limb dominance was previously determined using the lateral preference inventory (Coren, 1993). In order to calculate the total score (YBT), the distances between the furthest reaching positions of the boxes from the center were summed up. After six practice attempts for familiarization reasons (Schmidt et al., 2015), three recorded attempts were performed. The average of the two best attempts was used for further analyses. High reliability was previously reported for this testing procedure (ICC = 0.85–0.93, SEM: 2.0–3.5 cm) (Shaffer et al., 2013).

Quality of life was assessed using the validated German version of the SF-36 questionnaire (Kurth and Ellert, 2002). The test comprises 36 questions in 8 different health dimensions: physical functioning (PF), role limitations due to physical health problems (RP), bodily pain (BP), general health (GH), vitality (VT), social functioning (SF), role limitations due to emotional problems (RE), and mental health (MH). For each dimension a score ranging from 0 (maximum limitation) to 100 (minimal limitation) was calculated (Ware et al., 1993). Further, the mean of the four scores of PF, RP, BP, GH, and the mean of the four scores of VT, SF, RE, MH were calculated to provide a summary measure for the physical component of health (PCS) and mental component of health (MCS), respectively (Ware et al., 1993).

### Training procedures

All training sessions consisted of a standardized full-body warm-up (~5 min), followed by the resistance training program comprising four exercises conducted with the participants' own bodyweight as resistance (Table 2). The TRT group completed the resistance training program with a traditional set structure, consisting of 3 sets of 10 continuous repetitions and 90 s of rest between the sets. The CT group had a 20 s inter-repetition rest after every 3 repetitions, thus completing 10 series of 3 repetitions. Accordingly, both protocols were matched in terms of exercise selection, load, work-to-rest-ratio, and volume (Table 2). During the baseline measurements, participants were familiarized with all training exercises. During this familiarization period, all exercises were explained and demonstrated by an experienced strength and conditioning coach (M.L.). Subsequently, participants performed a few repetitions of each exercise under supervision and were provided with feedback. In order to find the right level of difficulty for each exercise, the various training exercises were completed at PRE under supervision of an experienced strength and conditioning coach (M.L.). The respective level at which 10 repetitions could be performed in a technically correct manner with a perceived effort of 5–6 on the 1–10 RPE scale was selected as the difficulty level for the respective exercise. During the intervention, the training stimulus was subsequently increased (i.e., exercise difficulty increased by one level) every 2 weeks to allow for a progressive training stimulus. After each session, the perceived effort (sRPE) on the 1–10 RPE scale was rated by every participant. For further analyses, the mean value of sRPE ratings for each session was calculated for all participants over the entire intervention period (mean sRPE).

**Table 2:** Six-week training protocol for both training groups.

**Exercise**	**Load** **(sets × repetitions)**	**Levels of difficulty**
Squat	CT: 10 ×3 TRT: 3 ×10	1) Sit-to-Stand on a standard chair 2) Sit-to-Stand on a standard chair without sitting 3) Squat with knee flexion <90° 4) Squat with knee flexion <90° and subsequent jump
Lunge (for both legs)	CT: 10 ×3 TRT: 3 ×10	1) Controlled flexion of front leg (~120°) 2) Controlled flexion of front leg (~90°) 3) Fast flexion of front leg (~90°) 4) Fast flexion of front leg (~90°) and subsequent jump
Crunch	CT: 10 ×3 TRT: 3 ×10	1) Legs in 90° flexion and placed on floor; flexion of the upper body while lumbar spine always maintains contact to floor; towel in both hands behind the thighs for support 2) Legs in 90° flexion placed on floor; flexion of the upper body while lumbar spine always maintains contact to floor 3) 90° flexion in the knees and hip (lower leg parallel to floor); flexion of the upper body while lumbar spine always maintains contact to floor 4) Fully extended legs held in air; flexion of the upper body while lumbar spine always maintains contact to floor
Plank	CT: 10 ×3 TRT: 3 ×10	1) On the floor in a ventral position, forearms flat on the ground, toes on the ground, knees on the ground; lifting both knees from the ground into full body extension 2) On the floor in a ventral position, forearms flat on the ground, toes on the ground; moving the body forward and backwards by alternating ankle joint extension/flexion 3) On the floor in a ventral position, forearms flat on the ground, toes on the ground; lifting the feet 10–15 cm alternating with fully extended knees 4) On the floor in a ventral position, forearms flat on the ground, toes on the ground; lifting the feet and contralateral arm 10–15 cm alternating with fully extended knees

For the Cluster-training group (CT), 9 × 20 s of inter-repetition-rest were granted; the traditional training group (TRT) had 2 × 90 s of inter-set rest.

### Statistics

The summary statistics of the variables are presented as mean ± SD. All variables were initially tested and verified for normal distribution via Shapiro-Wilk-tests and variance homogeneity. To examine baseline group differences (TRT vs. CT) for the respective outcome measures (YBT, LP, AP, OM-STS, and SF-36) univariate (one factor) repeated measures analyses of variance (rANOVA) were conducted. Furthermore, separately conducted 2 (mode: TRT vs. CT) × 2 (time: ΔRUN-IN vs. ΔINTERVENTION) rANOVA were calculated for each outcome measure. Effect sizes for rANOVA are provided as partial eta squared ($\eta_{\text{p}}^{2}$), with ≥0.01, ≥0.06, ≥0.14 indicating small, moderate, and large effects, respectively (Cohen, 1988). In case of significant interaction effects, Bonferroni *post-hoc* tests were subsequently computed. Further, in order to examine group differences in mean sRPE, an independent *t*-test was calculated. For pairwise effect size comparison, standardized mean differences (SMD) were calculated as differences between means divided by the pooled standard deviations (trivial: | SMD | < 0.2, small: 0.2 ≤ | SMD | < 0.5, moderate: 0.5 ≤ | SMD | < 0.8, large: | SMD | ≥ 0.8) (Cohen, 1988). Statistical analyses were performed using R in its integrated development environment RStudio (R Core Team, 2021). For all calculations, an α-level of 0.05 was used as threshold for statistical significance.

## Results

### Maximal voluntary contraction

**Figure 2:**
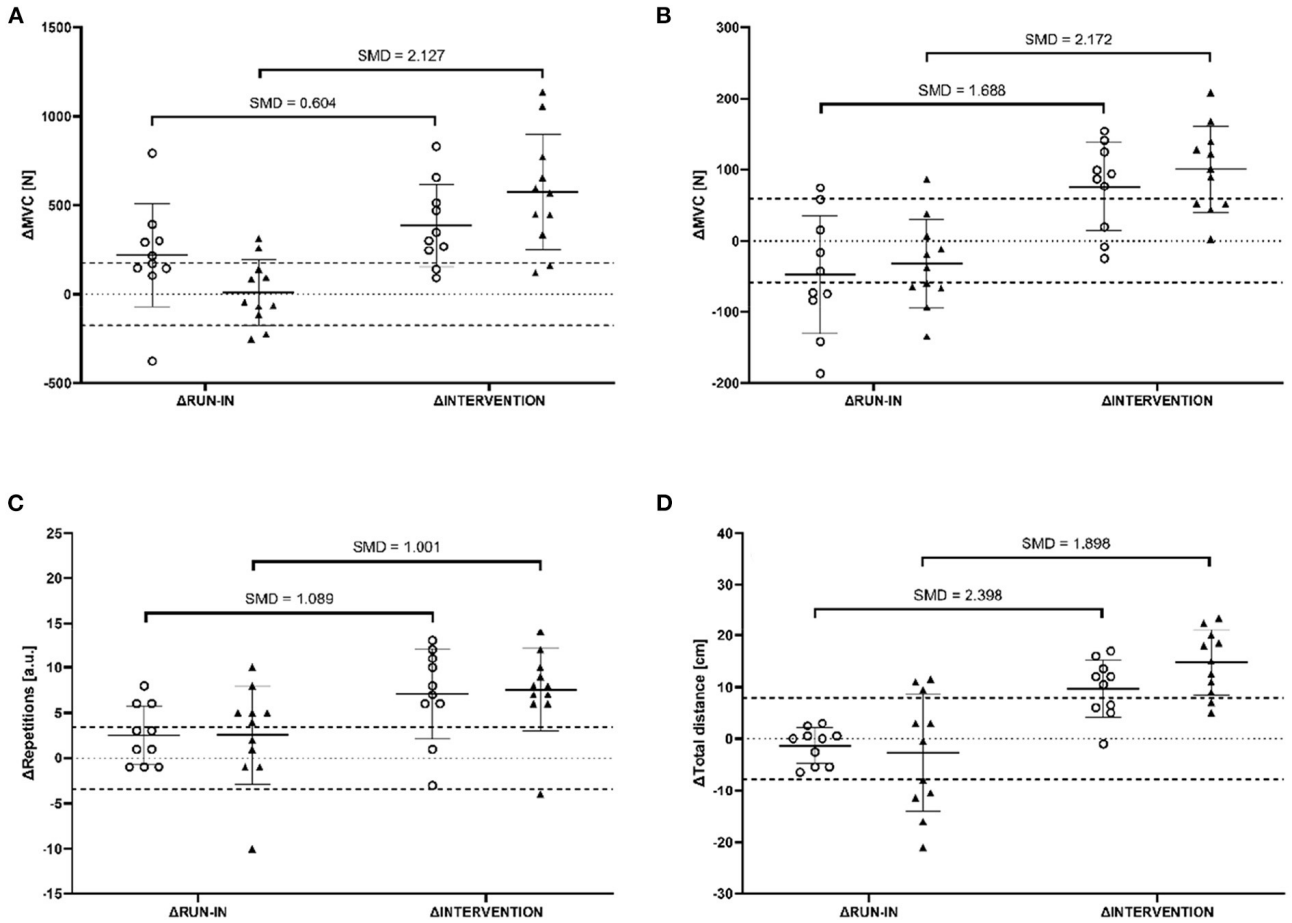
The difference between BASELINE and PRE (ΔRUN-IN) as well as PRE and POST (ΔINTERVENTION) of maximal voluntary contraction (MVC) of leg press machine **(A)**, MVC of abdominal press machine **(B)**, repetitions of one-minute-sit-to-stand **(C)**, and the reach distance of Y-Balance-test **(D)** for all participants of the cluster training group (circles) and traditional training group (triangles). Dashed horizontal lines indicate the smallest worthwhile change [30% of the populations' standard deviation at BASELINE (Hopkins, 2004)]. Further, mean values and standard deviations as well as standardized mean differences (SMD) are indicated.

For both LP and AP no significant mode × time interaction effect [*F*_(1, 19)_ = 4.225, *p* = 0.054, $\eta_{\text{p}}^{2}$ = 0.182 and *F*_(1, 19)_ = 0.043, *p* = 0.839, $\eta_{\text{p}}^{2}$ = 0.002, respectively], but significant and large main effects for time were observed [*F*_(1, 19)_ = 14.483, *p* = 0.001, $\eta_{\text{p}}^{2}$ = 0.433 and *F*_(1, 19)_ = 30.364, *p* < 0.001, $\eta_{\text{p}}^{2}$ = 0.615, respectively]. In LP, TRT showed remarkably greater improvements in ΔINTERVENTION compared to ΔRUN-IN (+572.6 ± 324.9 N vs. +10.5 ± 184.5 N, SMD = 2.127). For CT, greater improvements with moderate effects were found in ΔINTERVENTION compared to ΔRUN-IN (+386.2 ± 232.1 N vs. +218.3 ± 289.0 N, SMD = 0.640) (Figure 2A). In AP, both CT and TRT showed remarkably greater improvements in ΔINTERVENTION compared to ΔRUN-IN (+76.2 ± 61.7 N vs. −47.2 ± 83.0 N, SMD = 1.688 & +100.7 ± 60.5 N vs. −32.3 ± 61.9 N, SMD = 2.172, respectively) (Figure 2B).

### Dynamic strength endurance performance

For OM-STS, no significant mode × time interaction effect was observed [*F*_(1, 19)_ = 0.019, *p* = 0.891, $\eta_{\text{p}}^{2}$ = 0.001]. However, a simple main effect analysis of time [*F*_(1, 19)_ = 11.126, *p* = 0.003, $\eta_{\text{p}}^{2}$ = 0.369] revealed a significant and large effect, indicating remarkably greater improvements in ΔINTERVENTION compared to ΔRUN-IN (CT: +7.1 ± 5.0 a.u. vs. +2.5 ± 3.3 a.u., SMD = 1.089; TRT: +7.5 ± 4.6 a.u. +2.5 ± 5.4 a.u., SMD = 1.001) (Figure 2C).

### Functional balance performance

YBT revealed no significant mode × time interaction effect [*F*_(1, 19)_ = 1.776, *p* = 0.198, $\eta_{\text{p}}^{2}$ = 0.085]. However, a significant and large main effect for time [*F*_(1, 19)_ = 36.268, *p* < 0.001, $\eta_{\text{p}}^{2}$ = 0.656] was found, indicating remarkably greater improvements in ΔINTERVENTION compared to ΔRUN-IN (CT: +9.6 ± 5.6 cm vs. −1.4 ± 3.4 cm, SMD = 2.398; TRT: +14.7 ± 6.3 cm vs. −2.7 ± 11.0 cm, SMD = 1.898) (Figure 2D).

### Rating of perceived effort

**Figure 3:**
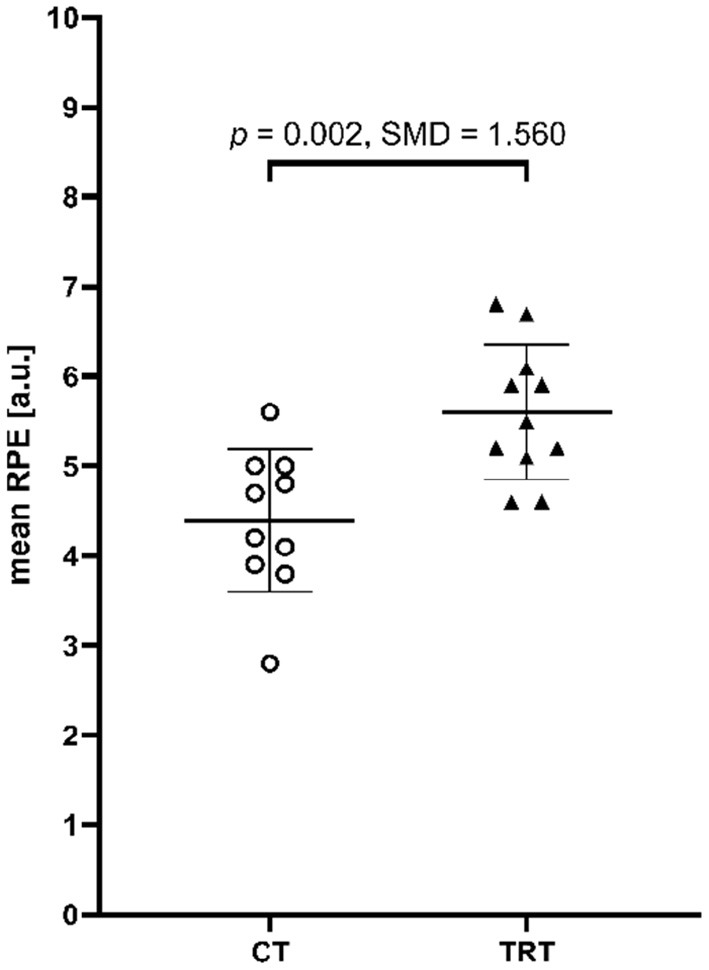
Mean perceived effort of each session for all participants of the cluster training group (CT, circles) and traditional training group (TRT, triangles). Further, mean values and standard deviations, *p*-values of the independent *t*-test, and standardized mean differences (SMD) are indicated additionally.

There was a significant effect for mean sRPE, with CT rating perceived effort lower than TRT [4.4 ± 0.8 vs. 5.6 ± 0.8 a.u., *t*_(19)_ = −3.569, *p* = 0.002, SMD = 1.560] (Figure 3).

### Quality of life assessment

For all dimensions of the SF-36, no significant mode × time interaction effects were observed (0.084 < *p* < 0.987, < 0.001 < $\eta_{\text{p}}^{2}$ < 0.149).

## Discussion

This randomized controlled trial aimed at investigating whether 6 weeks of homebased face-to-face online training conducted with a cluster training structure imposes similar strength and functional balance adaptations at a lower rate of perceived effort as traditional training matched in terms of exercise selection, load, volume, and work-to-rest-ratio in healthy, untrained middle-aged to older adults. Despite the lower reported perceived effort in the cluster structure training group, similar improvements in maximal voluntary contraction at the abdominal press machine, Y-Balance-test performance, and one-minute sit-to-stand test were observed for both training groups. Due to the run-in phase, these effects are likely to be induced by physical adaptations and not by familiarization effects of the tests. Interestingly, maximal voluntary contraction at leg press machine only improved statistically significant in the traditional training structure group. However, moderate improvements were also found for the cluster training structure group.

Our findings are in line with the results reported by Dias and colleagues (Dias et al., 2020). Dias and colleagues (2020) compared functional performance and physical fitness adaptations in postmenopausal and elderly women randomly assigned to either 12-weeks of volume-matched resistance training consisting of whole-body exercises with a traditional set structure (3 × 8 repetitions with 120 s of inter-set rest) or training with cluster set structure (12 × 2 repetitions with 30 s of inter-repetition rest) twice a week (Dias et al., 2020). In this study, significant improvements in 10 RM leg press and 30-second sit-to-stand test were found. However, the adaptations did not differ between the two training groups. In a similar study, Ramirez-Campillo et al. (2018) reported that functional performance (e.g., 10 m-walking speed test, 8-foot-up-and-go test, 30 s-sit-to-stand test) in elderly women showed statistically greater improvements after 12 weeks of high-speed resistance training with a cluster set structure (12 × 2 repetitions with 30 s of inter-repetition rest) compared to a volume-matched resistance training program executed with a traditional set structure (3 × 8 repetitions with 150 s of inter-repetition rest) conducted three times a week. Both studies were similar in exercise selection but differed in terms of training frequency (twice per week; Dias et al., 2020 vs. thrice a week; Ramirez-Campillo et al., 2018) and exercise order (alternating between exercises for the upper and lower body; Dias et al., 2020 vs. first exercises for the upper body, then for the lower body; Ramirez-Campillo et al., 2018). Therefore, it was hypothesized that exercise selection and order may impact the adaptations in favor of cluster training when the muscles of the lower body and upper body are trained consecutively rather than alternately. In this regard, less recovery time for each muscle group between the respective exercises in a traditional training setting compared to cluster training was mentioned as a possible factor (Dias et al., 2020). In the present study, however, both leg exercises were performed prior to core exercises. A lower training load (the participants' own bodyweight vs. machine-based training at 45, 60, and 75% 1 RM) and a lower training frequency (two vs. three times weekly) may account for the lack of significant differences in adaptations between traditional and cluster training in the present study compared to previous research (Ramirez-Campillo et al., 2018). On the other hand, in a volume-matched training setting, similar adaptations regarding strength and hypertrophy were found between training intensities ranging from 20 to 80% of 1RM (Lasevicius et al., 2018). Further, a 6-week volume-matched RCT indicated no differences in maximal strength adaptations between a training group training six times per week and a training group training three times per week (Colquhoun et al., 2018). Therefore, as both training groups in the present study were matched in terms of load, exercise selection, volume, and work-to-rest-ratio, similar adaptations in both groups seem plausible.

Strength training is known to increase neuromuscular activation (Aagaard et al., 2002). This may explain the increases in balance performance after strength training, as the central nervous system regulates muscular recruitment trough efferent signaling in moments of imbalance to regain a balanced position (Rogers et al., 2013). Hence, an increase in muscular strength of the core muscles improves balance performance (Granacher et al., 2013). Additionally, small, but significant correlations between lower extremity strength and dynamic/static balance performance have been reported (Muehlbauer et al., 2015). Therefore, our combination of strength training for the lower extremities and core partly under unstable conditions (lunge performed in tandem stance) may be seen as a sufficient stimulus to increase balance performance, hence explaining increases in Y-Balance-Test performance in both groups.

Muscle fatigue in the lower extremities and the trunk acutely impairs balance performance in the elderly (Helbostad et al., 2010) and is associated with a significant increase in the risk of falls (Helbostad et al., 2010; Nagano et al., 2014; Renner et al., 2021). Therefore, fatiguing exercise training is a potential risk factor for older persons in everyday life. In the present study, perceived effort was significantly higher in the traditional training group compared to the cluster training group. Similarly, in young, healthy sport science students, significantly higher perceived effort was reported after strength training conducted as repetition to failure (5 sets with a load of 10 RM, 3 min of rest between sets) compared to a load and work-rest-ratio matched cluster training (5 sets with a load of 10 RM, ~20–30 s of inter-repetition-rest) of bench press, but not squat (Mayo et al., 2014). Accordingly, Hardee et al. (2012) found significant differences in terms of perceived effort in resistance trained men after 18 repetitions of the power clean exercise performed in different set structures. In their study, 3 × 6 repetitions with 80% 1 RM were performed on different days in a randomized order with no, 20 s, and 40 s of inter-repetition-rest in addition to 3 min of rest between each set. While perceived effort did not differ between the training sessions conducted with a set structure of no and 20 s inter-repetition-rest, significantly lower levels of perceived effort were reported following the set structure with 40 s of inter-repetition-rest. In the present study, participants performed all exercises with their own body weight, which may induce lower overall levels of fatigue compared to training with additional load (Morishita et al., 2019). However, inter-repetition-rest of 20 s in the cluster training group was not granted after each repetition, but after bouts of three repetitions. Nevertheless, in a time- and load-matched setting (36 repetitions of back squat using a load of 75% 1 RM), existing research (Tufano et al., 2019) reported the rating of perceived effort not to differ between three different training sessions consisting of (I) 36 continuous repetitions with 12 s of inter-repetition-rest, (II) 9 bouts of 4 repetitions with 52.5 s of rest between bouts and (III) 9 bouts of 4 repetitions with 30 s of rest between bouts and an additional set rest of 120 s after the third and sixth bout. Therefore, it seems plausible that the lower rating of perceived effort in cluster training designs depends on the work-rest ratio. Thus, the duration of inter-repetition-rest should be chosen depending on the applied training load with an inter-repetition-rest of 20 s being sufficient in exercises performed with the own bodyweight.

A limitation of this study to be mentioned is the large variance in the age of our participants with individuals being aged between 40 and 70 years and the additional variance introduced by including both male and female participants. However, since (I) a sufficient technical understanding was required to participate in the online-based training sessions, (II) three measurement appointments had to be attended in the laboratory, (III) a higher drop-out rate due to the prevailing pandemic had to be expected, and (IV) the importance of cross-gender research, we decided to set the age limit at 40+ years to be able to include a sufficient number of participants of both sexes. The significant increase in strength in both training groups and the improvements in functional balance performance show that a homebased online training can induce similar adaptations as traditional non-homebased training. As resistance training has been shown to increase muscular quality regardless of age or sex (Ivey et al., 2000), these results may also be applied to older populations. Within an aging society (Lutz et al., 2008), the need for regular training to maintain physical health will increase in the future, and online training can be a tool to provide a live-instructed training independent of the location. This is especially the case as future generations of older people will have a sufficient technical understanding and will therefore access this kind of training easier. Furthermore, it should be noted, that participants' physical activity apart from the training sessions was not tracked. However, during a short interview at BASELINE the participants were asked to report their mean physical activity per week over the last 4 weeks and subsequently asked, to not alter their behavior in terms of physical activity during the study. Additionally, we employed the minimization method (Scott et al., 2002) and strived for minimal group differences for age, body mass index (BMI), sex, mean isometric strength test results, mean activity per week (in minutes), and initial level of exercise difficulty, thus keeping baseline differences between the training groups at a minimum.

In conclusion, isometric maximal strength, dynamic strength endurance and dynamic balance performance in healthy, untrained middle-aged to older adults can be significantly improved by 6 weeks of a homebased online live training conducted with a traditional training set structure as well as with a cluster set structure. However, perceived effort was rated lower by participants training with cluster set structure compared to participants of the load- and work-rest-ratio-matched traditional training group. Therefore, using a homebased online live training setting especially conducted with a cluster set structure can be considered a suitable approach for improving the physical performance of middle-aged to older persons.

## Data availability statement

The raw data supporting the conclusions of this article will be made available by the authors, without undue reservation.

## Ethics statement

The studies involving human participants were reviewed and approved by Ethical Committee of the German Sport University Cologne. The patients/participants provided their written informed consent to participate in this study.

## Author contributions

SH, LR, ML, and LD contributed to the conception and design of the study. ML led the intervention. LR and SH performed the statistical analysis. LR wrote the first draft of the manuscript. SH, PW, and LD wrote sections of the manuscript. PW copyedited the draft for content, language, and format and organized the submission and revision/resubmission process. All authors contributed to the article and approved the submitted version.

## Funding

We acknowledge the financial support of the German Research Foundation (DFG) and the Open Access Publication Fund of Bielefeld University for the article processing charge.

## Conflict of interest

The authors declare that the research was conducted in the absence of any commercial or financial relationships that could be construed as a potential conflict of interest.

## Publisher's note

All claims expressed in this article are solely those of the authors and do not necessarily represent those of their affiliated organizations, or those of the publisher, the editors and the reviewers. Any product that may be evaluated in this article, or claim that may be made by its manufacturer, is not guaranteed or endorsed by the publisher.
